# Dual-Ring SNAREpin Machinery Tuning for Fast Synaptic Vesicle Fusion

**DOI:** 10.3390/biom14050600

**Published:** 2024-05-19

**Authors:** Matthieu Caruel, Frédéric Pincet

**Affiliations:** 1 Univ Paris Est Creteil, Univ Gustave Eiffel, CNRS, UMR 8208, MSME, F-94010 Créteil, France; matthieu.caruel@u-pec.fr; 2 Laboratoire de Physique de l’École Normale Supérieure, ENS, Université PSL, CNRS, Sorbonne Université, Université Paris Cité, F-75005 Paris, France

**Keywords:** peripheral SNAREpins, accelerated fusion, membrane fusion, protein folding

## Abstract

During neurotransmission, neurotransmitters are released less than a millisecond after the arrival of the action potential. To achieve this ultra-fast event, the synaptic vesicle must be pre-docked to the plasma membrane. In this primed state, SNAREpins, the protein-coiled coils whose assembly provides the energy to trigger fusion, are partly zippered and clamped like a hairpin and held open and ready to snap close when the clamp is released. Recently, it was suggested that three types of regulatory factors, synaptophysin, synaptotagmins, and complexins act cooperatively to organize two concentric rings, a central and a peripheral ring, containing up to six SNAREpins each. We used a mechanical model of the SNAREpins with two separate states, half-zippered and fully zippered, and determined the energy landscape according to the number of SNAREpins in each ring. We also performed simulations to estimate the fusion time in each case. The presence of the peripheral SNAREpins generally smoothens the energy landscape and accelerates the fusion time. With the predicted physiological numbers of six central and six peripheral SNAREpins, the fusion time is accelerated at least 100 times by the presence of the peripheral SNAREpins, and fusion occurs in less than 10 μs, which is well within the physiological requirements.

## 1. Introduction

**Figure 1:**
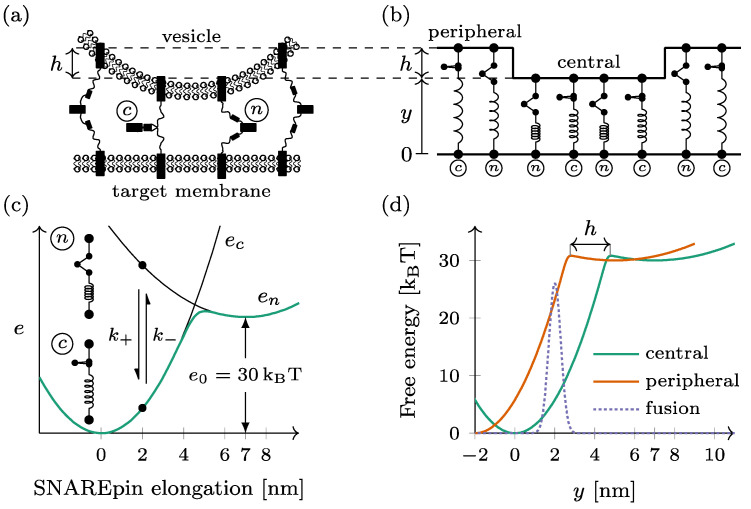
Mechanical model of the dual-ring SNAREpin machinery. (**a**) The SNAREpins form two rings called the central ring and the peripheral ring around the fusion point. (**b**) In each ring, the SNAREpins are arranged in parallel such that the intermembrane distance is constant within a given ring. The vertical shift between the two rings is denoted by h. (**c**) Each SNAREpin can be either half-zippered (*n*) or zippered (*c*). In each of the two states, the energy $e_{n,c}$ depends quadratically on the SNAREpin total elongation (black lines). The conformational change dynamic is a two-state jump process n⇌c, with rates $k_{+}$ and $k_{-}$. For a given SNAREpin elongation, free energy is a double-well potential (green line). (**d**) When represented as function of the central ring intermembrane distance y, the free energy of a peripheral SNAREpin (orange) is shifted by h compared to that of a central SNAREpin (green). The fusion repulsion forces derive from a Gaussian energy barrier (dashed line).

Membrane fusion is a widespread cellular process employed, for instance, in intracellular trafficking [1,2,3]. In this process, vesicles, ranging in diameter from 40 to 200 nm, fuse with a target membrane to release their cargo. The fusion process is not spontaneous due to an activation energy of approximately 30 k_B_T, necessitating external energy to trigger it [4,5,6,7,8]. This energy is derived from the assembly of a protein complex known as a SNAREpin, formed by the combination of four zipped, coiled coils [9,10,11]. SNAREpins pull the vesicle and target membranes together, compelling them to fuse. The zippering of SNAREpins typically takes several seconds to minutes, yet this slowness does not usually impede the efficiency of the molecular response to the external stimulus that triggers it [12]. For instance, hormone secretion can occur within minutes of the incoming signal. However, neurotransmission presents a unique case where timing is crucial. The rapid release of neurotransmitters from one neuron to another or a muscle must occur in less than 1 ms after the arrival of the action potential [13]. This rapid kinetics is incompatible with the slow assembly of SNAREpins. To address this challenge and expedite neurotransmitter delivery, SNAREpins between synaptic vesicles and target neuronal plasma membranes were partially assembled. The remaining zippering was obstructed by regulatory proteins, including synaptophysin, synaptotagmins, Munc13, and complexins. These bound vesicles form a “readily releasable pool” [14,15,16,17,18,19]. The intricate molecular structure formed by these regulatory factors and the events leading to its formation and disassembly are not fully elucidated. Nevertheless, recent results suggest the concentric radial distribution of two groups of SNAREpins around the vesicle–plasma membrane contact point: a ring of up to six central SNAREpins and a ring of up to six peripheral SNAREpins [20,21]. Synaptophysin would be responsible for templating pairs of SNAREpins, each pair containing a SNAREpin of the central ring and a SNAREpin of the peripheral ring. Complexins would help to clamp the SNAREpins and position the pair relative to each other. A synaptotamin ring-like oligomer, templated by Munc13, would sterically prevent fusion and be disrupted upon calcium entry. According to this hypothesis, the central SNAREpins were anchored approximately 8 nm away from the vesicle/plasma membrane contact point, while the peripheral SNAREpins were approximately 5 nm further away; see Figure 1a for a sketch of the geometry. The central SNAREpins delineate the area where the fusion pore opens. The presence of peripheral SNAREpins is believed to expedite fusion by accelerating the initial fusion pore opening (referred to as fusion time) and pressurizing the vesicle, thereby increasing the speed of fusion pore expansion and subsequent neurotransmitter release [21]. Our study focuses on the first aspect, fusion time, utilizing a mechanical model we previously developed to predict the energy landscapes leading to SNARE-induced fusion pore opening. We explored the impact of peripheral SNAREpins and revealed, as expected, a significant acceleration in fusion time. However, the results are contingent on both the position and actual number of SNAREpins in both the central and peripheral rings. We provide a comprehensive description, varying both the position of the peripheral ring relative to the central ring and the number of SNAREpins and present phase diagrams summarizing the influence of peripheral SNAREpins on the activation of the energy barriers.

## 2. Materials and Methods

### 2.1. Double-Ring Mechanical Model

The purpose of the model is to provide a mechanical representation of how the presence of two rings of SNAREpins induced by the cooperative action of synaptophysin, synaptotagmins, complexins, and Munc13 can affect the dynamics of SNAREpin zippering and synaptic vesicle fusion. The model is an extension of the one proposed in [22], in which the presence of synaptophysin and complexins was ignored, i.e., only the central ring of SNAREpin was considered. The model assumes that the fusion pore opens at the center of the SNAREpin rings and that, within the central ring, the two membranes are well represented by pure lipid bilayers. Hence, any function of synaptophysin, synaptotagmins, and/or complexins in controlling and timing vesicle fusion is not considered. These regulatory factors may very well slow down (e.g., by steric hindrance) or accelerate (e.g., by pressurization [21]) the fusion pore kinetics.

The docked dual-ring SNAREpin machinery consists of two ensembles of SNAREpins forming distinct rings, hereafter called the central and the peripheral rings, around the fusion point (see Figure 1a). Within each ring, the SNAREpins are arranged in parallel: the intermembrane distance is the same for all the elements within the same ring (see Figure 1b). The intermembrane distance of the central ring—closest to the target membrane—is denoted by y for this ring. For the SNAREpins constituting the peripheral ring—farther from the target membrane– the intermembrane distance is increased by a vertical shift h. The number of SNAREpins is denoted by $N_{c}$ and $N_{p}$ in the central ring and the peripheral ring, respectively.

Each SNAREpin is modeled as a spring that has two metastable states: the half-zippered state, where the N-terminal domain is zippered, and the fully zippered state, where the N- and C-terminals are both zippered. These metastable states are characterized by optical tweezers [23,24,25]. Physical quantities are denoted with index n when they refer to the half-zippered state and index c when they refer to the fully zippered state.

In both states, we assume that the force born by a SNAREpin is proportional to its elongation, which makes the energy $e_{n,c}$ of each state a quadratic function of the elongation. The relaxed configuration of the fully zippered state defines the origin of the elongation and serves as a reference for the energies. The relaxed configuration of the half-zippered state corresponds to an elongation of a=7 nm [22] and an energy offset of $v_{0}=30$ k_B_T.

Hence, the energy of a SNAREpin with elongation x is obtained by (Figure 1c)

$$\begin{array}{ll} e_{n}\left(x\right) & =\frac{\kappa_{n}}{2}{\left(x-a\right)}^{2}+v_{0}\;\mathrm{in}\;\mathrm{the}\;\mathrm{half}-\mathrm{zippered}\;\mathrm{state},\\ e_{c}\left(x\right) & =\frac{\kappa_{c}}{2}{\left(x\right)}^{2}\;\mathrm{in}\;\mathrm{the}\;\mathrm{zippered}\;\mathrm{state}. \end{array}$$

This elongation x is directly the intermembrane distance. x=y in the central ring, while it is obtained by x=y+h in the peripheral ring.

In thermal equilibrium, the free energy of the SNAREpin with a given elongation x is obtained by (see Figure 1c)

$$f_{s}\left(x\right)=-k_{B}Tlog\;\left\{exp\;\left[-e_{c}\left(x\right)/\left(k_{B}T\right)\;\right]+exp\;\left[-e_{n}\left(x\right)/\left(k_{B}T\right)\;\right]\right\}$$

When represented as a function of the intermembrane distance y, the free energy of a peripheral SNAREpin is shifted leftward with respect to the free energy of a central SNAREpin by the amount h (see Figure 1d).

The fusion is considered to be irreversible when the distance between the vesicle membrane and the target membrane at the level of the central ring falls below the critical value of $y_{f}=$2 nm (see Figure 1d). To reach this point, the system has to overcome the short-range repulsion forces between the two membranes. These forces are derived from a Gaussian energy barrier $e_{f}\left(y\right)=v_{f}exp\left[-{\left(y-y_{f}\right)}^{2}/\left(2\sigma_{f}^{2}\right)\right]$ (see the dotted line in Figure 1d).

Considering a system with $N_{c}$ central SNAREpins and $N_{p}$ peripheral SNAREpins, the total free energy of the system can be written as

$$f\left(y\right)=N_{c}f_{s}\left(y\right)+N_{p}f_{s}\left(y+h\right)+e_{f}\left(y\right).$$

### 2.2. Fusion Dynamics

The conformational change in a single SNAREpin is represented as a stochastic jump process n⇌c, with a forward rate $k_{+}$ and a reverse rate $k_{-}$ that depend on the elongation of the SNAREpin x.

Considering a SNAREpin with elongation x and introducing the characteristic zippering rate k, we postulate the following:

$$\begin{array}{rr} & k_{-}\left(x\right)=k\;\mathrm{and}\;k_{+}=kexp\left\{\left[e_{n}\left(x\right)-e_{c}\left(x\right)\right]/\left(k_{B}T\right)\right\},\;\mathrm{if}\;e_{n}\left(x\right)<e_{c}\left(x\right),\\ & k_{+}\left(x\right)=k\;\mathrm{and}\;k_{-}=kexp\left\{\left[e_{c}\left(x\right)-e_{n}\left(x\right)\right]/\left(k_{B}T\right)\right\},\;\mathrm{if}\;e_{n}\left(x\right)\geq e_{c}\left(x\right), \end{array}$$

which verifies the detailed balance condition $k_{+}/k_{-}=exp\left\{-\left[e_{c}\left(x\right)-e_{n}\left(x\right)\right]/\left(k_{B}T\right)\right\}$, where $k_{B}$ is the Boltzmann constant, and T is absolute temperature.

The zippering–unzippering dynamics can be simulated by considering that the probability for an individual SNAREpin to change its conformation within an interval $\left[t,t+\delta t\right]$ is obtained by $k_{\pm }\delta t$. We denote, by $c_{t}$ and $n_{t}$, the number of zippered SNAREpins in the central and peripheral ring, respectively. For the central ring, we define $P_{c}^{+}(t)$ (or conversely, $P_{c}^{-}(t)$) as the probability that one SNAREpin of this ring zippers (or conversely, unzippers) in the time interval $\left[t,t+\delta t\right].$ Similarly, we can define the same probabilities, namely, $P_{p}^{+}(t)$ and $P_{p}^{-}(t)$ for the peripheral ring.

At the first order in δt, we obtain:

$P_{c}^{+}(t)=\left(N_{c}-c_{t}\right)k_{+}(y)\delta t$ and $P_{p}^{+}(t)=\left(N_{p}-p_{t}\right)k_{+}(y+h)$ for the zippering probabilities and:

$P_{c}^{-}(t)=c_{t}k_{-}(y)\delta t$ and $P_{p}^{-}\left(t\right)=p_{t}k_{-}\left(y+h\right)\delta t$ for the unzippering probabilities.

The motion of the vesicle is considered in the overdamped regime. It is driven by the force exerted by the SNAREpins from the two rings, the repulsive force between the membrane, the viscous interaction with the surrounding fluid, and thermal forces. The force exerted by the two rings of SNAREpins depends on the intermembrane distance and the number of zippered SNAREpins $c_{t}$ and $p_{t}$ in the central and peripheral ring, respectively. The dynamics of the intermembrane distance resulting from the balance between these forces can be written as the following Langevin stochastic differential equation

(1) $$\eta \;dy_{t}=-\partial_{y}\left[e\left(y_{t},c_{t},p_{t}\right)+e_{f}\left(y_{t}\right)\right]dt+\sqrt{2\eta \;k_{B}T}dB_{t},$$

where η is a drag coefficient, and $dB_{t}$ is a Brownian motion increment drawn from normal distribution with standard deviation $\sqrt{dt}$. The force exerted by the SNAREpins derives from the potential

$$e\left(y,c,p\right)=c\;e_{c}\left(y\right)+\left(N_{c}-c\right)e_{n}\left(y\right)+p\;e_{c}\left(y+h\right)+\left(N_{p}-p\right)e_{n}\left(y+h\right).$$

As initial conditions, we considered the primed state preceding the activation of synaptic vesicle fusion by Ca^2+^ ion entry. The modeled primed state is characterized by y=7 nm and c=p=0. For a given realization, the simulation ends when $y<y_{f}$, i.e., when fusion occurs.

The calibration is similar to the one used in [22], to which we referred for more details about its methodology. The parameter values are indicated in Table 1.

### 2.3. Simulations

The simulations were performed using a fixed timestep of 9 ps. At each timestep, the states of the SNAREpins ensembles were updated according to an acceptation–rejection algorithm for each ring. For the central ring, the algorithm is (i) draw a uniformly distributed number r ∈[0, 1] and (ii) if $r<P_{c}^{+}(t)\;then\;c_{t+\delta t}=c_{t}+1,\;else\;if\;r<P_{c}^{+}(t)+P_{c}^{-}(t)\;then\;c_{t+\delta t}=c_{t}-1\;else\;c_{t+\delta t}=c_{t}$. A similar algorithm was used to compute $p_{t+\delta t}$.

Once the new configuration of the rings was known, the position of the vesicle was updated according to Equation (1) using the explicit Euler–Maruyama method. More details about the simulation methods can be found in the Supplementary Materials of Ref. [22]. Averages were computed from 10^3^ realizations.

The results were computed using a custom Julia (v 1.10.2) program [26].

The figures were generated using the pgfplot LaTeX package (v 1.18).

### 2.4. Fusion Kinetics in a Single-Ring Setting

**Figure 2:**
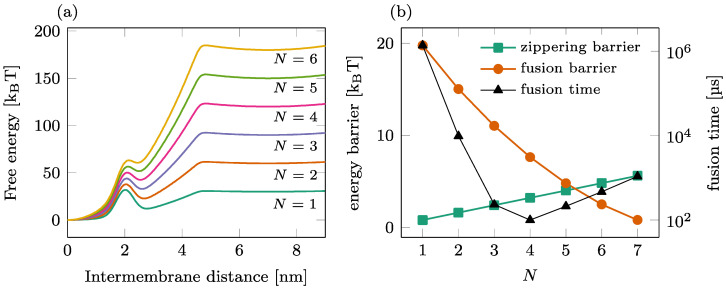
Fusion process in a single-ring setting. (**a**) Overall free energy of the reduced one-dimensional model for different numbers of SNAREpins. (**b**) Influence of the number of SNAREpins on the fusion kinetics. Left axis: value of the energy barriers for zippering (intermembrane distance ~4.5 nm) and fusion (intermembrane distance ~2 nm); right axis: average waiting time before fusion.

The fusion process mediated by a single ring of a SNAREpin is illustrated in Figure 2. The total free energy landscape is represented for various numbers of SNAREpins N in Panel (a), and the corresponding zippering and fusion barriers are shown in Panel (b) (left axis). As N increases, the fusion barrier reduces (circles) as more SNAREpins can exert more force on the two membranes. In contrast, the zippering barrier increases (squares) with N, showing that the synchronization of the individual zippering events requires more time for large groups of SNAREpins. A detailed analysis of this phenomenon can be found in [27]. The result of these antagonist dependences is the optimal number of SNAREpins, leading to an overall average fusion time of ~100 μs, as presented in [22].

## 3. Results

Here, we reiterated the analysis of an effective energy landscape previously performed with a single-ring setting and applied it to the case of a double-ring setting. The following presents a parametric study focusing on the characteristics of the double-ring setting in terms of (i) the vertical shift between the two rings; (ii) the number of SNAREpins in the peripheral ring, and (iii) the number of SNAREpins in the central ring.

### 3.1. Effect of the Vertical Shift between the Two Rings

**Figure 3:**
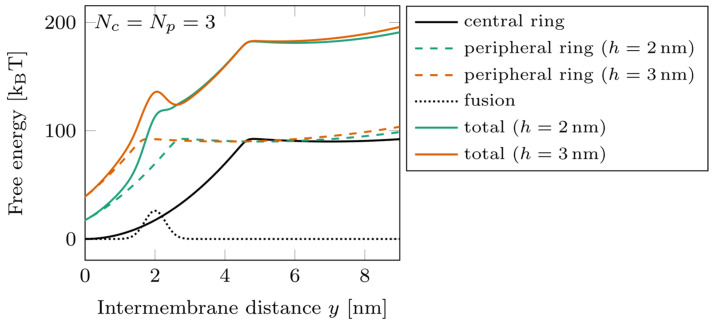
Energy landscape of a double-ring system, with $N_{c}=N_{p}=3$ at two different ring shifts. Dotted line: fusion barrier; solid black line: central ring free energy; dashed lines: peripheral ring free energy h=2nm (green) and h=3nm (orange); solid colored lines: total free energy for h=2 nm (green) and h=3 nm (orange).

The energy landscapes characterizing the system are shown in Figure 3 for $N_{c}=N_{p}=3$, with two different offset values, h=2 nm (green) and h=3 nm (orange). A slight change in the vertical shift between the rings can have a strong effect on the free energy barrier corresponding to the fusion. Switching the position of the peripheral ring from h=2 nm to h=3 nm displaces its zippering transition point beyond the position of the barrier. In such a case, the peripheral ring remaining mostly unzippered tends to separate the membranes by a distance of about 4 nm and, therefore, tends to slow down the fusion kinetics. This effect is the basis of the results of our parametric study.

**Figure 4:**
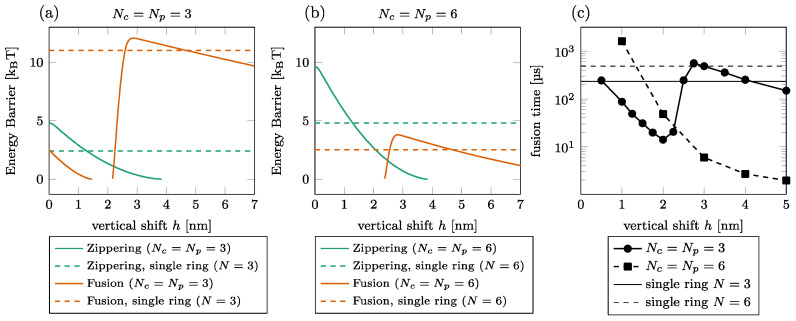
Effect of vertical shift between the rings. [(**a**,**b**)] Zippering (green) and fusion (orange) energy barriers for $N_{c}=N_{p}=3$ (**a**) and $N_{c}=N_{p}=6$ (**b**). The graph interruptions signal configurations where the barrier does not exist. The horizontal lines show the barriers in a single-ring setting. (**c**) Fusion time for $N_{c}=N_{p}=3$ (solid, circles) and $N_{c}=N_{p}=6$ (dashed, squares). The thin horizontal lines show the fusion time in a single-ring setting for N=3 (solid) and N=6 (dashed).

The effect of the shift on the fusion time is illustrated in Figure 4 for $N_{c}=N_{p}=3$ and $N_{c}=N_{p}=6$. We first observed that the barriers do not necessarily exist for all vertical shifts h as the graph interruptions in (a) and (b) signal. For instance, the zippering barrier is removed beyond h=4 nm of the shift for $N_{c}=N_{p}=3$; see (a) solid green line. The main observation is that the dependence of the fusion energy barrier on the vertical shift is non-monotone, showing a steep increase by a few k_B_T between h=2 and h=3 nm, especially in the case of $N_{c}=N_{p}=3$. Consequently, the fusion time also increases in this interval by around one order of magnitude for $N_{c}=N_{p}=3$ [circles in (c)]. We do not observe this effect for $N_{c}=N_{p}=6$ since the increase of the fusion barrier is less pronounced; see (b).

If we compare the energy barrier with and without the peripheral ring (dashed vs. solid lines in Figure 4), we find that for the low vertical shift, the presence of the second ring is detrimental to the zippering process. The fusion process is, however, almost always facilitated by the peripheral ring, except in a short interval around h=3 nm, where the fusion energy barrier is larger with the peripheral ring than without.

### 3.2. Effect of the Number of SNAREpins in the Peripheral Ring

**Figure 5:**
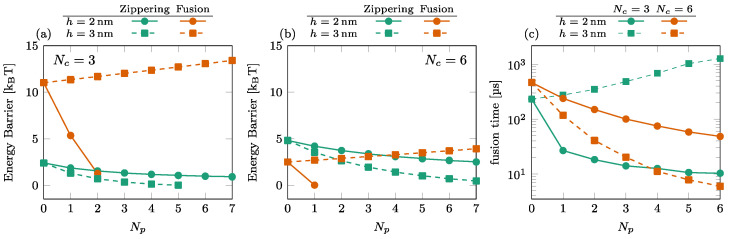
Effect of the number of SNAREpins in the peripheral ring. [(**a**,**b**)] Energy barriers for $N_{c}=3$ (**a**) and $N_{c}=6$ (**b**). Zippering (green) and fusion (orange) barriers are shown for both h=2nm (solid lines, circles) and h=3nm (dashed lines, squares). Interrupted curves signal the absence of the corresponding barriers. (**c**) Fusion time for $N_{c}=3$ (green) and $N_{c}=6$ (orange) for both h=2nm (solid lines, circles) and h=3nm (dashed lines, squares).

We illustrate the effect of the number of SNAREpins in the peripheral ring in Figure 5. The energy barriers are shown for $N_{c}=3$ (a) and $N_{c}=6$ (b). These results again show the importance of the relative positioning of the rings. For h=2 nm (solid disks), the fusion barrier is a rapidly decreasing function of $N_{p}$, while for h=3 nm (orange squares), it increases with $N_{p}$. Consequently, the time to cross the fusion barriers increases with the number of SNAREpins in the peripheral ring; see, for instance, the case of $N_{c}=3$ and h=3 nm in Figure 5c.

### 3.3. Effect of the Number of SNAREpins in the Central Ring

**Figure 6:**
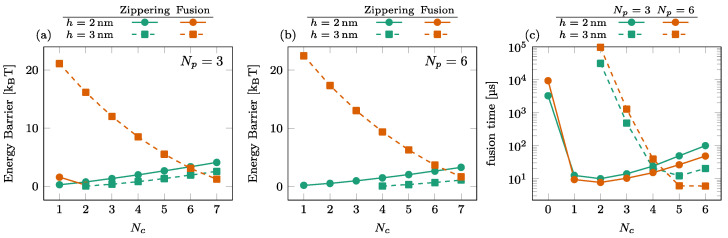
Effect of the number of SNAREpins in the central ring. [(**a**,**b**)] Energy barriers for $N_{p}=3$ (**a**) and $N_{p}=6$ (**b**). Zippering (green) and fusion (orange) barriers are shown for both h=2nm (solid lines, circles) and h=3nm (dashed lines, squares). Interrupted curves signal the absence of the corresponding barriers. (**c**) Fusion time for $N_{p}=3$ (green) and $N_{p}=6$ (orange) for both h=2nm (solid lines, circles) and h=3 nm (dashed lines, squares).

The dependence of the energy barriers and the fusion time on the number of SNAREpins in the central ring $N_{c}$ is illustrated in Figure 6. The effect of $N_{c}$ is most noticeable in the fusion barrier for h=3 nm [orange squares in (a) and (b)], showing a rapid decrease with $N_{c}$. For h=2 nm, the fusion barrier is cancelled by the addition of one or two SNAREpins in the peripheral ring [circles in Figure 5a,b]. In this case, the effect of $N_{c}$ is visible only in the increase of the zippering barrier. The consequence on the overall time of fusion is the rapid decrease observed with the addition of a single central SNAREpin corresponding to the disappearance of the fusion barrier and a subsequent slower increase in the fusion time due to the progressive increase of the zippering barrier with $N_{c}$; see circles in Figure 6c.

For h=3 nm, the addition of peripheral SNAREpins increases the fusion barrier [squares and circles in Figure 5a,b], and it requires more SNAREpins in the central ring to counterbalance this effect. The minimum fusion time is then reached at $N_{c}\approx 5$ with h=3 nm, while it is reached at $N_{c}\approx 2$ with h=2 nm.

### 3.4. Summary of the Energy Barriers and Fusion Time

**Figure 7:**
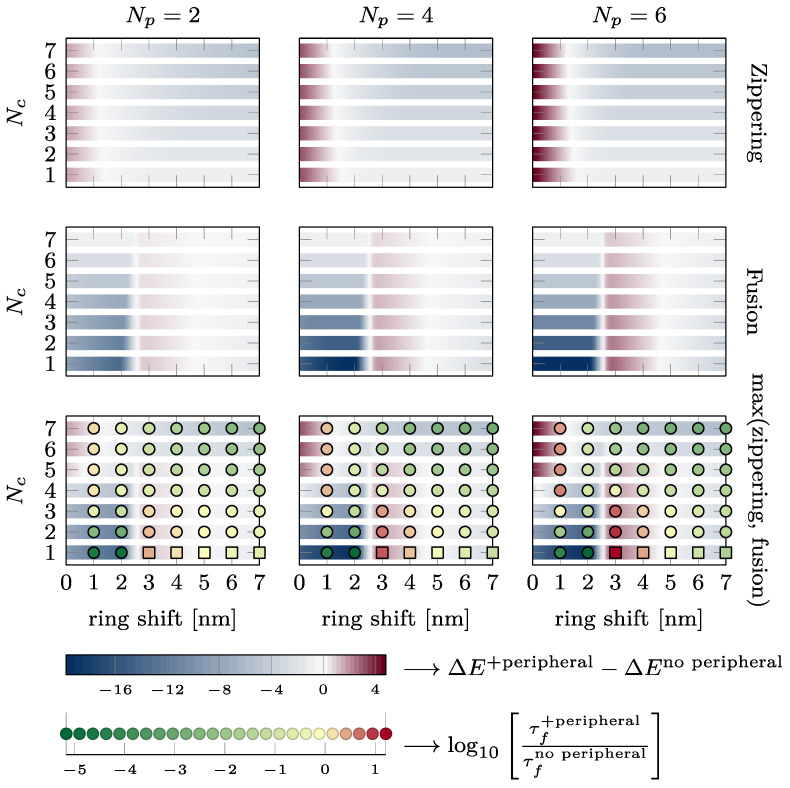
Effect of the presence of the peripheral ring on zippering, fusion energy barriers, and fusion time. Each plot shows the intervals of h, where the presence of the peripheral ring reduces (see blue intervals) or increases (see dark red intervals) the energy barriers. The different lines of plots correspond to different energy barriers from first to third line: zippering barrier, fusion barrier, and the maximum of these two barriers. On the third line, the disks and squares indicate whether the fusion time is accelerated (green) or slowed down (red). Disks are obtained from simulations, whereas squares are estimated from Kramers approximation since the simulations are too long to finish in a reasonable time. The two bottom lines provide the color code. The variations in energy are reported in k_B_T, and the changes in fusion are represented on logarithmic scale (base 10).

The effect of the presence of the external ring on the energy barriers characterizing the kinetics of the fusion process and the fusion time is summarized in Figure 7. The two upper lines show the change in energy of the zippering (first line) and fusion barrier (second line) in k_B_T. A blue color indicates that the energy barrier is reduced by the peripheral SNAREpins. A dark red color indicates that the energy barrier is increased by the peripheral SNAREpins. The last line shows the change in the maximum energy barrier in the total energy landscape and the change in fusion time on a logarithmic scale. Green disks indicate accelerated fusion time, and red disks correspond to slower fusion times. It is worth noting that increasing one of the barriers may still lead to a more favorable energy landscape and accelerated fusion times because the other barrier is reduced enough. For instance, for five central and six peripheral SNAREpins, which is close to the expected physiological values, and a vertical shift of 3 nm, the fusion barrier is increased by approximately 4 k_B_T, but the maximum barrier of the overall energy landscape is reduced by 1 k_B_T, and the fusion time is accelerated 100 times.

Observation of the first two rows of Figure 7 shows that the presence of the peripheral SNAREpins reduces the zippering energy barrier as soon as h>1.5 nm. The fusion barrier is reduced except near h=3 nm.

If we now consider the maximum between the zippering and fusion barriers, we find that for $N_{c}<4$, the fusion energy barrier is larger than the zippering energy barrier and is, therefore, likely to impose the overall fusion kinetics.

## 4. Discussion

### 4.1. Role of the Vertical Shift

As depicted in Figure 7, for the almost the whole range of vertical shift, *h*, between the central and peripheral rings, the presence of peripheral SNAREpins significantly accelerates the fusion time. This acceleration primarily occurs by smoothing the energy landscape of the central SNAREpins alone, thereby reducing both zippering and fusion barriers. In terms of forces, peripheral SNAREpins act to pull the membranes together when the central SNAREpins are unable to exert any pulling force.

In spite of this beneficial effect of peripheral SNAREpins on fusion, there are two detrimental regions in the phase diagram of Figure 7 that exhibit a counterproductive action, slowing down the fusion process.

First, when there are five or more central SNAREpins, and *h* is below 1.5 nm, the zippering energy barrier increases with the number of peripheral SNAREpins, leading to a longer fusion time. This increase comes from the small value of *h*, where the positions of the peripheral SNAREpins closely resemble those of the central SNAREpins, effectively making them function as central SNAREpins. Consequently, the fusion process is similar to the scenario predicted for a central SNAREpin ring alone, as displayed in Figure 2b (black line). Hence, beyond three SNAREpins, including both the central and peripheral, the fusion time increases with the number of SNAREpins because of the increase in the zippering of the energy barrier.

The second region where the presence of the peripheral SNAREpins is unfavorable lies between the h values of 2.5 and 4.5 nm, with fewer than six central SNAREpins. This counterintuitive observation arises from the zippering energy barrier separating the half- and fully zippered state of the peripheral SNAREpins located approximately 3.5 nm from the fusion barrier; see Figure 1d. Hence, when the vertical shift is close to 3.5 nm, peripheral SNAREpins exert a repulsive force that raises the fusion barrier, instead of facilitating fusion by smoothing the energy landscape.

### 4.2. Energy Barrier vs. Fusion Time

The last row of Figure 7 allows a direct comparison of the energy barriers and the fusion time. In most cases, favorable energy barriers (in blue) match accelerated fusion times (in green), and vice versa, unfavorable energy barriers (dark red) are correlated with slower fusion times (red). Hence, energy barriers that are straightforward to compute from energy landscapes, such as the one presented in Figure 3, are a good proxy to predict whether the fusion time will increase or decrease. The few cases in which the energy barrier does not correctly predict the change in the fusion time correspond to the energetically unfavorable values of the vertical shift (between 2.5 and 4.5 nm). In these cases, the fusion time is actually accelerated up to two orders of magnitude. Hence, even though the energy landscape may seem unfavorable, the presence of the peripheral SNAREpins accelerates fusion.

### 4.3. Physiological Consequences

In the model proposing the existence of central and peripheral SNAREpin rings, it is hypothesized that each ring comprises six SNAREpins. According to the bottom right panel of Figure 7, with this specific number of central and peripheral SNAREpins, the fusion time accelerates for any vertical shift exceeding 1.5 nm. The predictions suggest that the diameters of the central and peripheral rings are approximately 15 nm and 25 nm, respectively [21]. For a 40 nm vesicle, these dimensions position the central and peripheral rings approximately 1.5 nm and 4.4 nm above the bottom of the vesicle, as depicted in Figure 1, Panel (a). Hence, within this model, the vertical shift would be of the order of 3 nm, which is sufficient to ensure that the system operates beyond the first detrimental region where the peripheral SNAREpins impede the fusion process. However, if the vesicle fails to provide four central SNAREpins, the calculated value of the vertical shift indicates that the system will fall into the second detrimental region. Hence, according to this model, it is critical that precisely six or more central SNAREpins are formed, while the number of peripheral SNAREpins remains less critical.

## 5. Conclusions

Based on our model, it is clear that the inclusion of peripheral SNAREpins can drastically hasten the opening of the fusion pore. We selected values of the parameters for the energy landscapes that align with the experiment data. While these parameters may not be entirely precise, and the quantitative descriptions provided here may not be absolutely accurate, the fundamental features will persist despite variations in the parameter values. First, the vertical shift is expected to exceed 2 nm, indicating a distinct energy landscape for peripheral SNAREpins separate from that of the central SNAREpins. This implies non-overlapping zippering barriers between the two rings. Second, approximately six central SNAREpins are indispensable for accelerated fusion pore opening in the presence of peripheral SNAREpins. Third, a higher count of peripheral SNAREpins correlates with a swifter initial fusion pore opening. For example, in the proposed physiological scenario of six central SNAREpins, six peripheral SNAREpins, and a vertical shift of 3 nm, we anticipate the initial opening of the fusion pore occurring 50 μs after the release of the clamp compared to 1 ms without peripheral SNAREpins, marking a 200-fold acceleration.

## Figures and Tables

**Table 1:** Parameters of the model. FB*: fusion barrier. For details about the calibration methodology, we referred to [22].

Parameter	Symbol	Value	Unit
Zippering distance	a	7	nm
Energy bias	$v_{0}$	30	k_B_T
Unzippered stiffness	$\kappa_{n}$	1.5	pNnm^−1^
Zippered stiffness	$\kappa_{c}$	12	pNnm^−1^
FB* position	$y_{f}$	2	nm
FB width	$\sigma_{f}$	0.3	nm
FB energy	$v_{f}$	26	k_B_T
Drag coefficient	η	$3.8\times 10^{-7}$	Nsm^−1^

## Data Availability

The data are contained within the article and the Supplementary Materials.

## Supplementary Materials

The following supporting information can be downloaded at: https://www.mdpi.com/article/10.3390/biom14050600/s1, Figure S1: Website for the automatic determination of the energy landscapes and energy barriers with the number of SNAREpins and the vertical shift.
