# Supplementary material for: Dual-Ring SNAREpin Machinery Tuning for Fast Synaptic Vesicle Fusion
Source: Biomolecules. 2024 May 19;14(5):600. doi: 10.3390/biom14050600 (PMC11117985; doi:10.3390/biom14050600)
Supplement: Supplementary file 1 [file biomolecules-14-00600-s001.zip › biomolecules-2972658-supplementary.pdf]

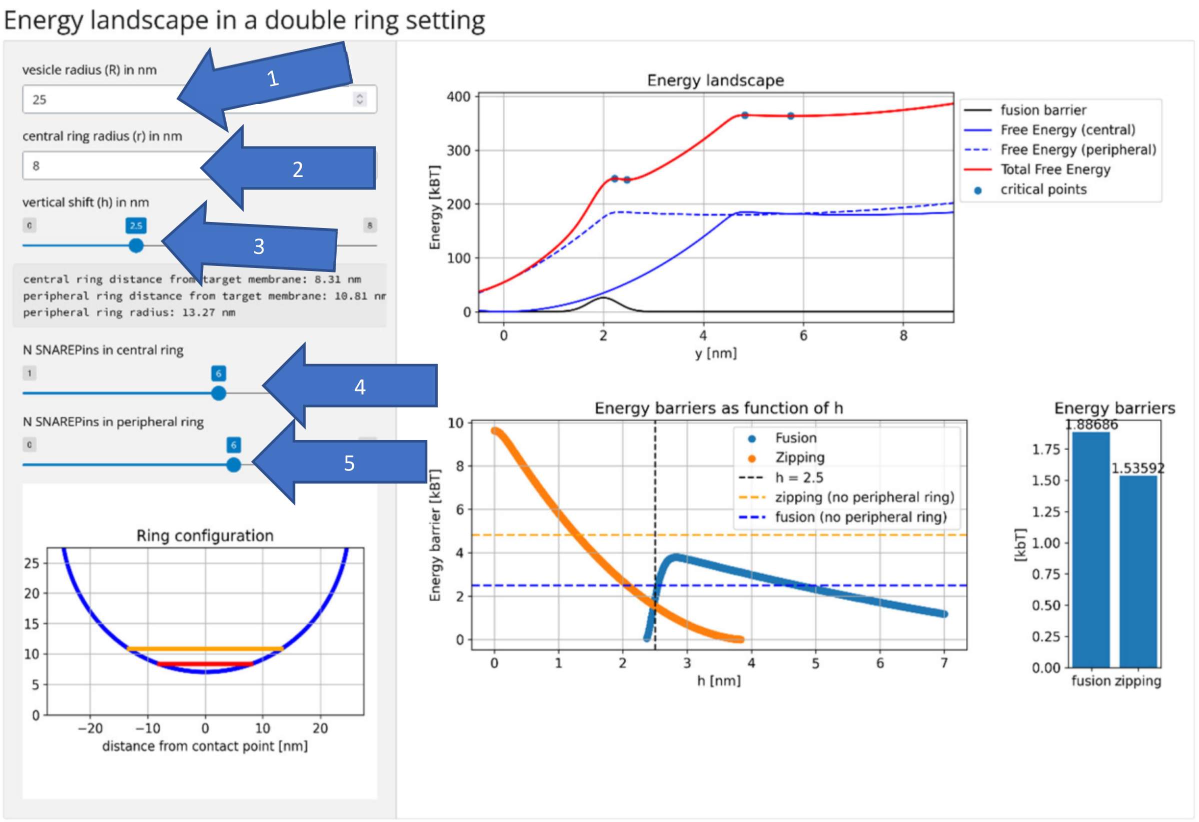

Figure S1. Website for the automatic determination of the energy landscapes and energy barriers with the number and SNAREpins and the vertical shift. We have written a code to automatically calculate the energy landscape and energy barriers. The corresponding application can be found in the second zip file. Clicking on this link will prompt to a window like the one in the snapshot presented here. Five inputs indicated by the blue arrows are required: 1 is the radius of the vesicle in nm; 2 is the radius of the central ring; 3 is the vertical shift; 4 is the number of SNAREpins in the central ring; 5 is the number of SNAREpins in the peripheral ring. Once these parameters are set, the text in the middle of the left column, and the 4 panels (bottom left, central column and bottom right) are automatically adjusted. Please be patient as it sometimes takes a few second for the update. In the snapshot, parameters close to the hypothesized physiological ones were chosen: 1. 25 nm radius vesicle; 2. 8 nm radius for the central SNAREpin; 3. 2.5 nm vertical shift; 4. 6 central SNAREpins; 5. 6 peripheral SNAREpins. With these parameters, the text in the middle of the left column indicates that the SNAREpins in the central ring are initially stretched by 8.31 nm, the SNAREpins in the peripheral ring are initially stretched by 10.81 nm and the radius of the peripheral ring is 13.27 nm. The bottom left panel indicates the location of the central (red) and peripheral (orange) rings. The top middle panel shows the free energy of the central (blue) and peripheral (dashed blue) SNAREpins, the fusion barrier (black) and the total free energy that includes the central and peripheral rings and fusion barrier (orange). The bottom middle panel displays the fusion (blue) and zipping (orange) energy barriers for vertical shifts between 0 and 7 nm. The blue (resp. orange) dashed line indicates the value of the fusion (resp. zipping) energy barrier in the absence of peripheral SNAREpins with the same number of central SNAREpins as considered in the parameters. The vertical black dashed line indicates the vertical shift considered in the parameters. The bottom left panel provides the values of the fusion and zipping energy barriers in k_BT. When there is no barrier, the corresponding column is removed from the plot.
