# Supplementary material for: Dual-Ring SNAREpin Machinery Tuning for Fast Synaptic Vesicle Fusion
Source: Biomolecules. 2024 May 19;14(5):600. doi: 10.3390/biom14050600 (PMC11117985; doi:10.3390/biom14050600)
Supplement: Supplementary file 1 [file biomolecules-14-00600-s001.zip › Second Zip.zip!/supplementary-material.pdf]

Dual-Ring SNAREpin Machinery Tuning for Fast Synaptic Vesicle Fusion

Supplementary Material

Matthieu Caruel Frédéric Pincet

Content

This supplementary material provides the source code that generates an interactive Python `shiny app`. This app to interactively observe the the effects of the model parameters N_c, N_p and h on the energy landscapes of the system and its barriers.

For more information about `shiny apps` we refer to the shinylive website.

The downloaded `.zip` file contains the following:

- `css` directory: contains a minimal `.css` stylesheet
- `app.py`: the code of the shiny app
- `barriers_data.csv`: the dataset of the energy barriers for the considered ranges of parameters values
- `manifest.json`: metadata of the application
- `requirements.txt`: to set the Python environment in which the app was developed

Execution procedure

This app was developed using Python v3.11.5.

1. Install `shiny`
2. Open a terminal in the directory where `app.py` is located
3. Execute the command: `shiny run --reload app.py`
4. The command will provide a local URL like `http://127.0.0.1:8000` that can be pasted in a web browser to use the app locally
